# Multi-objective optimization of enzyme manipulations in metabolic networks considering resilience effects

**DOI:** 10.1186/1752-0509-5-145

**Published:** 2011-09-19

**Authors:** Wu-Hsiung Wu, Feng-Sheng Wang, Maw-Shang Chang

**Affiliations:** 1 Department of Computer Science and Information Engineering, National Chung Cheng University, Chiayi 62102, Taiwan; 2 Department of Chemical Engineering, National Chung Cheng University, Chiayi 62102, Taiwan

## Abstract

**Background:**

Improving the synthesis rate of desired metabolites in metabolic systems is one of the main tasks in metabolic engineering. In the last decade, metabolic engineering approaches based on the mathematical optimization have been used extensively for the analysis and manipulation of metabolic networks. Experimental evidence shows that mutants reflect resilience phenomena against gene alterations. Although researchers have published many studies on the design of metabolic systems based on kinetic models and optimization strategies, almost no studies discuss the multi-objective optimization problem for enzyme manipulations in metabolic networks considering resilience phenomenon.

**Results:**

This study proposes a generalized fuzzy multi-objective optimization approach to formulate the enzyme intervention problem for metabolic networks considering resilience phenomena and cell viability. This approach is a general framework that can be applied to any metabolic networks to investigate the influence of resilience phenomena on gene intervention strategies and maximum target synthesis rates. This study evaluates the performance of the proposed approach by applying it to two metabolic systems: *S. cerevisiae *and *E. coli*. Results show that the maximum synthesis rates of target products by genetic interventions are always over-estimated in metabolic networks that do not consider the resilience effects.

**Conclusions:**

Considering the resilience phenomena in metabolic networks can improve the predictions of gene intervention and maximum synthesis rates in metabolic engineering. The proposed generalized fuzzy multi-objective optimization approach has the potential to be a good and practical framework in the design of metabolic networks.

## Background

Improving the synthesis rate of desired metabolites in metabolic systems is one of the main tasks in metabolic engineering. Two recent advancements in this area are promising to increase the performance of metabolic systems. The first factor is a significantly better understanding of the structure of metabolic networks and the kinetics and thermodynamics of biochemical reactions that take place in living cells. In many cases, this understanding is not merely qualitative but quantitative, and can be expressed in terms of kinetics equations. The second factor is the current advance in molecular biological techniques and the development of numerous useful vectors. This has enabled microbiologists to change the protein content in a given organism and alter its enzymatic profile, enhancing the synthesis of specific end-products or intermediates. The combination of these two factors permits the modification of the metabolic structure and the improvement of the synthesis rate of some desired metabolites in an organism.

In the last decade, many researchers have used model-based optimization strategies to analyze and manipulate metabolic networks [1-12]. The mathematical models used in these model-based optimization problems can be classified as stoichiometric and kinetic models. Stoichiometric models can be obtained through the reaction topology of a metabolic network. Though stoichiometric models do not require kinetic data and are easy to construct, there is a shortage of handling regulatory dynamics of metabolic networks in them. On the other hand, kinetic models, e.g., generalized mass action (GMA) and Michaelis-Menten formulations, require more information to describe system characteristics. However, kinetic models are in general expressed as nonlinear models that are more complex than linear models and require more computational time for analysis and optimization. Logarithmic transformation can convert a non-linear model represented by the S-system formalism used widely in biochemical systems theory (BST) to a linear model if we consider systems at steady state only [7,12]. Indirect optimization methods (IOMs) convert a nonlinear kinetic model into an S-system model, and then solve the optimization problem at steady state using a linear programming method [10-13]. On the contrary, stochastic optimization methods and deterministic branch-and-reduce methods are directly applied to nonlinear models to obtain a global optimum [4,15]. Optimization problems for metabolic network systems can be categorized as single-objective and multi-objective formulations, depending on the design purpose. Most studies on microbial metabolic engineering focus on only a single objective to maximize the synthesis rate of the desired metabolite [4,16]. In contrast, a multi-objective optimization approach attempts to find the solutions that are optimal for many objectives simultaneously. The multi-objective indirect optimization method (MOIOM) has been applied to maximize ethanol productivity and to minimize intermediate concentrations simultaneously [10].

Selecting a proper genetic manipulation strategy for metabolic network optimization problem is a tedious task. The regulatory structure of metabolic networks can be determined by model-based optimization strategies [4,16]. Researchers have used mixed-integer linear programming to determine an optimal regulatory structure and the synthesis rate of metabolic systems described by linear models [4,5]. However, the minimum set of enzymes (or corresponding genes) in a metabolic system that should be manipulated to obtain a viable strain under the situation of producing the maximum possible flux or yield of a desired final product remains unclear. This study introduces a multi-objective optimization formulation to find an optimal regulatory structure to cope with these problems. Experimental results show that a strain may reflect resilience phenomenon after stressful environmental changes and genetic perturbations [17,18]. This resilience phenomenon means that the mutant strain may respond with rapid and dramatic alterations to global genetic perturbations. However, after genetic perturbations, the mutant tries to evolve to a new steady state that may be only slightly different from its previous steady state. This new steady state indicates that the mutant strain tries to recover from its "wild-type" characteristics and maintain relative stability on metabolism. Accurately predicting the steady state of a microbial strain after gene manipulations is not a trivial job, since the adaption of metabolic systems against gene alterations is complex. Segrèt et al. introduced the minimization of metabolic adjustment (MOMA) method to calculate the minimum distance solution relative to the original "wild-type" solution for the mutant strain [17]. Shlomi et al. applied regulatory on/off minimization (ROOM) to determine minimum number of changes between the mutant strain and the original strain [18]. However, both of these models are based on the stoichiometric model derived from the flux balance analysis (FBA). Almost no studies discuss the resilience phenomena for metabolic systems described by kinetic models.

This study introduces a generalized fuzzy multi-objective optimization problem (GFMOOP) to determine the optimal enzymatic manipulations for metabolic network systems considering resilience effects. This study first formulates a multi-objective optimization problem that simultaneously considers the resilience effects and minimum set of manipulated enzymes by combining the concepts of MOMA and ROOM into an optimization framework. Since nonlinear kinetic models offer a more detailed description of metabolic networks than stoichiometric models and the gene manipulations, including gene repressions and over-expressions, in metabolic networks can directly correspond to the changes of maximum flux parameters and reaction rates in the kinetic models, this study uses a nonlinear kinetic model in the optimization formulation. Integer variables are also introduced to model gene over-expression and repression. Thus, the optimization formulation must be solved using mixed-integer nonlinear programming (MINLP) methods. The metabolic networks for ethanol production by *Saccharomyces cerevisiae *and amino acid synthesis rates in *Escherichia coli *were employed to evaluate the applicability of the GFMOOP. Suitable membership functions are used to quantify the resilience effects and cell viability constraints. Results show that the maximum synthesis rates of target products by genetic interventions are always over-estimated in metabolic networks that do not consider the resilience effects.

## Results and discussion

Each following example solves two optimization problems and compares their results. The first problem is a primal optimization problem for determining the optimal enzyme manipulations, corresponding gene over-expression or repression, in metabolic networks without considering cell viability and metabolic adjustment. The second problem is the fuzzy optimization problem, which is similar to the primal optimization problem, but considers cell viability and metabolic adjustment.

### Maximization of the ethanol production by *S. cerevisiae*

**Figure 1:**
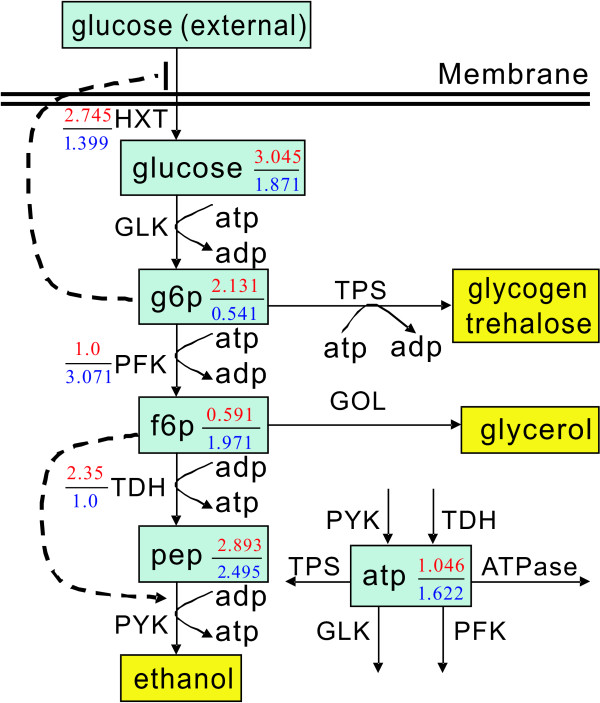
**Simple metabolic network of *S. cerevisiae *for ethanol production**. The dashed line with an arrowhead and with a terminal bar at one end mean inhibition and activation, respectively. The optimal changed ratios of the metabolites and the optimal improved activity ratios for modulated enzymes HXT and TDH and modulated enzymes HXT and PFK are shown in red numbers and blue numbers, respectively.

*S. cerevisiae *is still the most important microorganism for ethanol production to date. Researchers have developed many strategies to enhance ethanol productivity using yeast, and its metabolic network is well studied. Figure 1 shows a scheme of the simple metabolic network of *S. cerevisiae *for anaerobic ethanol production. Curto et al. developed a GMA model for analyzing the anaerobic ethanol fermentation of *S. cerevisiae *at steady state [19]. This model consists of five nonlinear ordinal differential equations and eight nonlinear rate equations. Detailed information about this model can be found in the Additional File 1.

**Figure 2:**
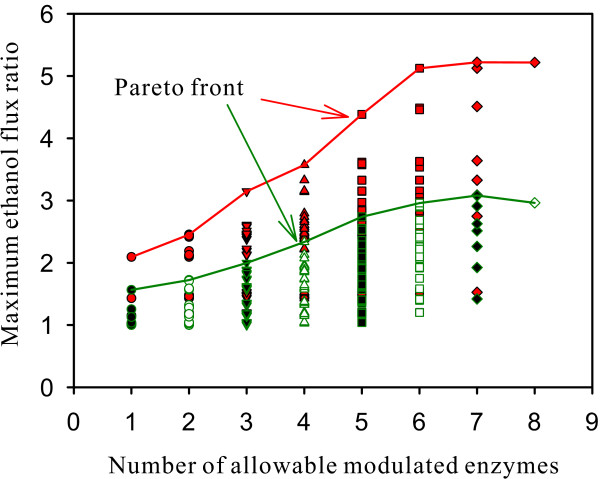
**The Pareto front and feasible solutions**. The Pareto front and feasible solutions for the primal optimization problem (red data points) and the fuzzy optimization problem (green data points) obtained by the MIHDE method.

This study uses the mixed-integer hybrid differential evolution (MIHDE) method [20,21] and the commercial software GAMS 23.6 with seven solvers to solve the ethanol fermentation optimization problem for finding the optimal enzyme manipulations in *S. cerevisiae*. The feasible region for each metabolite and enzyme can be estimated through biological understanding or global optimization techniques [22,23]. This study sets the feasible region for each metabolite and enzyme to expand/shrink 5-fold based on its basal value. The primal optimization problem for maximizing the ethanol productivity in *S. cerevisiae *was first solved by MIHDE to obtain the Pareto front, shown as the red curve in Figure 2. The larger the allowable number of the manipulated enzymes in the metabolic network, the higher the improved ethanol flux ratio, $v_{PYK}∕v_{PYK}^{basal}$. The highest improvement (about 5.2) was achieved when the allowable number of the manipulated enzymes was greater than six. Figure 2 shows all feasible solutions (red data points) for the primal optimization problem. Many improvements in the ethanol flux ratio are close to the highest value; for example, if at most two enzymes can be modulated, seven out of 28 feasible solutions with the improved ethanol flux ratio greater than 2.0 are obtained. The highest improved ratio is 2.452 and the corresponding modulated enzymes are HXT and PFK in this case.

This study also uses seven MINLP solvers in GAMS to solve the primal optimization problem with various allowable numbers of manipulated enzymes. Table 1 lists the maximum ethanol flux ratio and modulated enzymes using different allowable numbers of manipulated enzymes. These Pareto optimal solutions are identical to those obtained from the MIHDE method (Figure 2). However, some solvers may converge to a premature result, as shown in the parentheses of Table 1. The optimal solution obtained from the GAMS solvers can be improved by using the convergent solution obtained by MIHDE as the initial point for the GAMS solvers. However, it is time-consuming for MIHDE to obtain a feasible solution. The computational time increases significantly when we apply MIHDE to solve the primal optimization problem for a complex system. This is because it is difficult to maintain a feasible solution in the stochastic evolution procedure. The following computations apply combined procedures based on the seven GAMS solvers to determine an optimal solution. Two solvers were randomly selected from the seven solvers for each combined procedure. The convergent solution of the first solver served as the initial point for the second solver in each combined procedure. Seven different combined procedures were performed to determine the optimal solution for the primal optimization problem by comparing their optimal objective values. The results obtained by these combined procedures suggest that the enzymes to be modulated for the primal optimization problem are {HXT, PFK, PYK, TDH, GLK, ATPase, GOL, TPS} in decreasing order of priority (Table 1).

**Table 1:** The optimal solution for maximizing ethanol productivity by S. cerevisiae

*ε*-value	$v_{PYK}^{*}∕v_{PYK}^{basal}$	Modulated enzymes
1	2.092	HXT

2	2.452	HXT, PFK
	2.434 (SBB)^†^	HXT, ATPase

3	3.152	HXT, PFK, PYK

4	3.592	HXT, PFK, PYK, TDH
	3.326 (LINDOGlobal, BARON)^†^	HXT, PFK, PYK, ATPase

5	4.428	HXT, PFK, PYK, TDH, GLK

6	5.191	HXT, PFK, PYK, TDH, GLK, ATPase
	4.458 (DICOPT)^†^	HXT, PFK, PYK, TDH, GLK, GOL

7	5.231	HXT, PFK, PYK, TDH, GLK, ATPase, GOL
	3.651 (DICOPT)^†^	HXT, PFK, PYK, TDH, ATPase, GOL, TPS

8	5.231	HXT, PFK, PYK, TDH, GLK, ATPase, GOL, TPS

The optimal enzymatic modulations for maximizing ethanol productivity by *S. cerevisiae *obtained by solving the primal multi-objective optimization problem using various GAMS solvers (AlphaECP, BARON, BONMIN, COUENNE, DICOPT, LINDOGlobal, and SBB). $\gamma_{x_{i}}^{LB}$ and $\gamma_{e_{i}}^{LB}$ are set to 0.2. $\gamma_{x_{i}}^{UB}$ and $\gamma_{e_{i}}^{UB}$ are set to 5.0. The superscript * means optimal solution, † denotes that the solution is a premature result, and *ε *is the number of allowed manipulated genes.

Table 2 shows the results for the resilience problem, namely the fuzzy optimization problem considering resilience effects. The Pareto front of the resilience problem is identical to the result obtained by MIHDE, as shown in the green data curve of Figure 2. The maximum ethanol flux ratio for different allowable numbers of manipulated enzymes fell to 60-70%. The best enzymes appear to be glucose uptake (HXT) and glyceraldehyde-3-phosphate dehydrogenase (TDH) if only two enzymes can be modulated. The maximum ethanol flux of 1.71 was obtained by modulating HXT and TDH. These modulated enzymes obtained from the resilience problem differ from those obtained from the primal optimization problem (HXT and PFK). We also solve the resilience problem using the fixed enzyme modulations on HXT and PFK, which is a nonlinear programming (NLP) problem, for comparison. The optimal ethanol flux ratio of the NLP problem was 1.618, which is smaller than that obtained by modulated on HXT and TDH (Table 2). The ethanol synthesis flux, *v_PYK_*, was activated by the concentrations of metabolites, [f6p] and [pep], and inhibited by [atp] so that the improved ratio of *v_PYK _*to its basal value can be expressed as

**Table 2:** The optimal solution for maximizing ethanol productivity by S. cerevisiae considering resilience effects

*ε*-value	$v_{PYK}^{*}∕v_{PYK}^{basal}$	Modulated enzymes
1	1.482	HXT

2	1.710	HXT, TDH
	1.618^†^	HXT, PFK
	1.519^†^	HXT, ATPase

3	1.991	HXT, TDH, ATPase
	1.877^†^	HXT, TDH, PFK
	1.663^†^	HXT, PFK, PYK

4	2.340	HXT, TDH, PFK, PYK

5	2.741	HXT, TDH, PFK, PYK, GLK

6	3.080	HXT, TDH, PFK, PYK, GLK, ATPase

7	3.106	HXT, TDH, PFK, PYK, GLK, ATPase, GOL

8	3.105	HXT, TDH, PFK, PYK, GLK, ATPase, GOL, TPS

The optimal enzymatic modulation for maximizing ethanol productivity by *S. cerevisiae *considering cell viability and metabolic adjustment for $\left[\zeta_{x∕e}^{LB},\zeta_{x∕e}^{UB}\right]=\left[1.6,2.0\right]$. $\gamma_{x_{i}}^{LB}$ and $\gamma_{e_{i}}^{LB}$ are set to 0.2. $\gamma_{x_{i}}^{UB}$ and $\gamma_{e_{i}}^{UB}$ are set to 5.0. The superscript * means optimal solution, † indicates that the optimal result is obtained by solving fuzzy optimization problem using the fixed enzymatic modulations, and *ε *is the number of allowed manipulated genes.

(1) $$\begin{array}{lll} \frac{v_{PYK}}{v_{PYK}^{basal}} & ={\left(\frac{\left[f6p\right]}{{\left[f6p\right]}_{basal}}\right)}^{0.05}\times {\left(\frac{\left[pep\right]}{{\left[pep\right]}_{basal}}\right)}^{0.533}\times \; & \text{(1)}\\ & \;{\left(\frac{\left[atp\right]}{{\left[atp\right]}_{basal}}\right)}^{-0.0822}.\; & \text{(2)}\\ & \; & \text{(3)} \end{array}$$

The exponents in equation (1) indicate that the ethanol synthesis flux, *v_PYK_*, increases when the concentrations of metabolites [f6p] or [pep] increase and the concentration of metabolite [atp] decreases. Figure 1 shows the optimal flux ratios for the modulated enzymes HXT and TDH (red numbers) and modulated enzymes HXT and PFK (blue numbers), respectively. Figure 1 also shows the changed concentration ratios of the metabolites for the corresponding modulated enzymes. For the allowable modulated enzyme set {HXT, TDH}, the optimal concentration ratios of [f6p] and [atp] are smaller than those obtained by modulating enzymes HXT and PFK. This indicates that lower [atp] and higher [pep] increase the ethanol flux rate *v_PYK_*. This result makes sense from a biological viewpoint, since a lower [atp] level slows down cell growth and allows yeast to carry out the anaerobic fermentation required to produce ethanol. Although the exponent of [f6p] is positive, the small value causes an insignificant effect on the improved ethanol flux ratio. As a result, the maximum value of *v_PYK _*obtained by modulating enzymes HXT and TDH exceeds that by modulating enzymes HXT and PFK. Following the similar procedures, the best selected enzymes to be modulated for the resilience problem when at most three enzymes can be modulated are HXT, TDH, and ATPase. These results differ from those obtained from the primal optimization problem, as Tables 1 and 2 indicate. The ATPase enzyme does not appear in the suggested modulated enzyme set obtained from the resilience problem when four and five manipulated enzymes are allowed. As a result, we cannot prioritize the selection of enzymatic modulations in the optimization problems when at most three enzymes can be modulated. However, both primal optimization and resilience problems have the identical selected enzymes when the allowable number of manipulated enzymes is greater than three.

**Figure 3:**
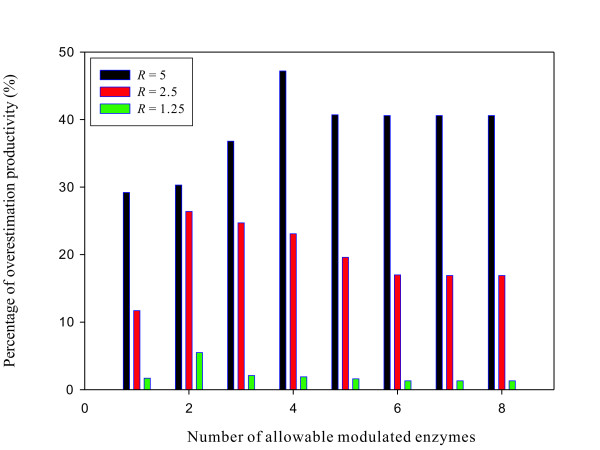
**Percentage of over-estimation productivity for different perturbation region**. The percentage of over-estimation productivity for different scale perturbation. The perturbation region for each enzyme is selected as *R*-fold below and above its basal value, i.e., $\left[\gamma_{e_{i}}^{LB},\gamma_{e_{i}}^{UB}\right]=\left[1∕R,R\right]$.

Each rate equation contains some metabolite concentration information so that the kinetic model is able to account for important regulatory features. Such regulations depend on the model validity because model parameter values are estimated from a normal condition. As a result, the kinetic model can cope with dynamics of metabolic networks under a small perturbation on enzyme level. However, an optimal enzyme manipulation problem may yield an over-estimation productivity if a large-scale change for each enzyme level is allowed. For instance, this study sets the perturbation region for each enzyme to expand/shrink 5-fold based on its basal value. Such a large-scale perturbation may reach beyond the regulation capability of kinetic models. The resilience phenomena can be applied to compensate such a gap. We also solve the primal and resilience optimization problems using two another smaller perturbation regions for each enzyme, i.e., the lower and upper factors $\left[\gamma_{e_{i}}^{LB},\gamma_{e_{i}}^{UB}\right]$ are set to [0.4, 2.5] and [0.8, 1.25], to illustrate the effect of resilience phenomena on optimal productivity under different perturbation regions. Figure 3 shows the percentage of over-estimation productivity for different number of allowable modulated enzymes and different perturbation region. For each number of allowable modulated enzymes, larger perturbation region results in higher over-estimation productivity. This result indicates that the resilience phenomena can be applied to compensate the regulation capability of kinetic models. It is worthy to verify this result experimentally by microbiologists.

### Multi-objective maximization of amino acid synthesis rates in *Escherichia coli*

**Figure 4:**
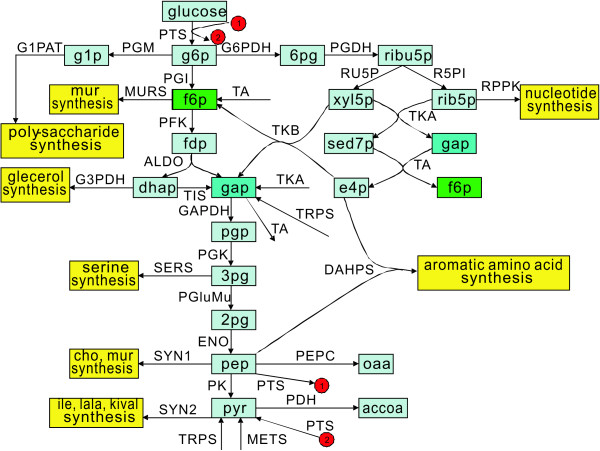
**Central carbon metabolic network of *Escherichia coli***. The light blue boxes are metabolites and the yellow boxes are amino acid synthesis subsystems. Red circles with a number inside are used for connection.

This example considers the determination of the optimal enzyme manipulation in the central carbon metabolic network of *E. coli*. The kinetic model for the network is complex and highly nonlinear. Chassagnole et al. developed a nonlinear dynamic model for part of central carbon metabolism of *E. coli *[24]. Their model has the ability to describe the experimentally observed dynamic behavior of metabolites in metabolic networks and is also capable of describing the intracellular metabolite oscillations observed in experiments [25]. This model links the kinetics of sugar transporter PTS (phosphor-transferase system) with glycolysis and pentose-phosphate pathways, and is used to support the exploration of the central carbon metabolism of *E. coli*. Figure 4 presents a schematic diagram of central carbon metabolism of *E. coli*. It depicts 30 enzymatic reactions, 18 metabolites or precursors, and seven co-metabolites (amp, adp, atp, nadp, nadph, nad, and nadh). These co-metabolite concentrations are assumed to be constant in the mathematical model. The reaction rates can be accessed from the model database of JWS Online Cellular Systems Modeling (http://jjj.biochem.sun.ac.za/). The detail information of the kinetic model for the central carbon metabolism in *E. coli *can be found in the Additional File 2.

Many researchers have applied optimization methods to enhance synthesis capabilities of microbial strains [16,26,27]. Most of the works on optimization of microbial strains focused on single-objective optimization. For example, Vital-Lopez et al. used a kinetic model of the central carbon metabolism of *E. coli *to identify optimal intervention strategies under the maximization of serine synthesis [16]. Lee et al. applied bi-objective optimization methods to investigate the influences of gene interventions on the amino acid synthesis using *E. coli *[26]. Lee et al. were interested in maximizing DAHPS, PEPC, and SERS enzymatic flux ratios that correspond to the enhancement of synthesis of aromatic amino acids, serine, and oxaloacetate, respectively. Their study solves two bi-objective optimization problems for maximizing DAHPS and PEPC flux ratios and maximizing DAHPS and SERS flux ratios, respectively, to determine the optimal gene manipulation strategies. However, this current study determines the optimal enzyme manipulation strategies to maximize the flux ratios of DAHPS, PEPC, and SERS simultaneously through genetic manipulations.

The procedures described in the previous example were again used to obtain the optimal Pareto solutions for the primal optimization problem with various allowable numbers of the manipulated enzymes (Table 3). The resilience problem was then solved using the lower and upper restriction factors of 1.6 and 2.0, respectively, for both fuzzy cell viability constraints. Table 4 shows the optimal enzymatic manipulations with various allowable numbers of the manipulated enzymes for the resilience problem. The optimal modulated enzymes for both optimization problems are the same as those in Tables 3 and 4. However, each maximum flux ratio for the resilience problem is smaller than that obtained from the corresponding primal optimization problem.

**Table 3:** The optimal solution for multi-synthesis maximization by E. coli

*ε*-value	$\frac{v_{PEPC}^{*}}{v_{PEPC}^{basal}}$	$\frac{v_{SERS}^{*}}{v_{SERS}^{basal}}$	$\frac{v_{DAHPS}^{*}}{v_{DAHPS}^{basal}}$	Modulated enzymes	**Optimal objective value **$(\eta_{D}^{*})$
1	1.271	1.081	1.560	PK	0.970
	1.342	1.068	1.780	G6PDH	0.975

2	1.248	1.518	1.652	G6PDH, SERS	0.846
	1.211	1.409	1.456	PK, SERS	0.878^†^
	1.778	1.106	2.185	PK, G6PDH	0.969

3	1.578	1.860	2.027	G6PDH, PK, SERS	0.782
	1.388	1.730	1.872	G6PDH, SERS, RPPK	0.814^†^

4	1.801	1.973	2.225	G6PDH, PK, SERS, RPPK	0.778
	1.492	1.934	2.175	G6PDH, PK, SERS, DAHPS	0.787^†^

5	1.597	2.258	2.467	G6PDH, PK, SERS, RPPK, DAHPS	0.763
	1.958	2.134	2.322	G6PDH, PK, SERS, RPPK, SYN1	0.786

The optimal enzymatic modulation to maximize aromatic amino acid, serine, and oxaloacetate synthesis rates simultaneously by *E. coli *without considering cell viability constraints. $\gamma_{x_{i}}^{LB}$ and $\gamma_{e_{i}}^{LB}$ are set to 0.2. $\gamma_{x_{i}}^{UB}$ and $\gamma_{e_{i}}^{UB}$ are set to 5.0. The superscript * means optimal solution, † denotes that the solution is not a Pareto solution, and *ε *is the number of allowed manipulated genes.

**Table 4:** The optimal solution for multi-synthesis maximization by E.coli considering resilience effects

*ε*-value	$\frac{v_{PEPC}^{*}}{v_{PEPC}^{basal}}$	$\frac{v_{SERS}^{*}}{v_{SERS}^{basal}}$	$\frac{v_{DAHPS}^{*}}{v_{DAHPS}^{basal}}$	Modulated enzymes	**Optimal objective value **$(\eta_{D}^{*})$
1	1.262	1.079	1.545	PK	0.971
	1.342	1.068	1.780	G6PDH	0.975

2	1.214	1.447	1.586	G6PDH, SERS	0.867
	1.186	1.365	1.407	PK, SERS	0.891^†^
	1.763	1.105	2.174	PK, G6PDH	0.968

3	1.443	1.740	1.884	G6PDH, PK, SERS	0.811
	1.314	1.591	1.764	G6PDH, SERS, RPPK	0.849^†^

4	1.582	1.829	2.044	G6PDH, PK, SERS, RPPK	0.810
	1.412	1.782	1.985	G6PDH, PK, SERS, DAHPS	0.821^†^

5	1.479	2.010	2.177	G6PDH, PK, SERS, RPPK, DAHPS	0.809
	1.704	1.980	2.143	G6PDH, PK, SERS, RPPK, SYN1	0.815

The optimal enzymatic modulation to maximize aromatic amino acid, serine, and oxaloacetate synthesis rates simultaneously by *E. coli *considering fuzzy cell viability constraints and fuzzy metabolic adjustment for .  and  are set to 0.2.  and  are set to 5.0. The superscript * means optimal solution, † denotes that the solution is not a Pareto solution, and *ε *is the number of allowed manipulated genes.

The optimal enzyme PK is selected from the 30-enzyme network if only one enzyme can be manipulated. The improved flux ratios of PEPC, SERS, and DAHPS for the primal optimal problem are nearly identical to those obtained from the resilience problem. This indicates that the resilience phenomenon has little effect on the cell response when only one enzyme alteration is allowed. In contrast, the optimal selected enzymes are G6PDH and SERS if two enzyme manipulations are allowed. Both flux ratios of SERS and DAHPS are enhanced even though the PEPC flux ratio is smaller than that obtained by modulating PK only. As a result, the pair of the selected enzymes (G6PDH and SERS) is a Pareto optimal solution. To confirm this result, solve the primal optimization problem and resilience problem using the fixed modulation pairs of (PK, SERS) and (PK, G6PDH). The three maximum flux ratios obtained by modulating PK and SERS are less than those obtained by manipulating G6PDH and SERS. This indicates that the modulation pair of (PK, SERS) is dominated by (G6PDH, SERS). The maximum flux ratios of PEPC and DAHPS increase and the maximum flux ratio of SERS decreases in comparison with those obtained by using the modulation pair (G6PDH, SERS). Thus, the result for the modulation pair (PK, G6PDH) is also a Pareto solution. However, the optimal objective value for (PK, G6PDH) exceeds that of manipulating G6PDH and SERS, so we obtain a single Pareto solution. The Pareto solution for manipulating G6PDH is similar to those show in Tables 3 and 4.

Similar procedures were also used to obtain the optimal modulations for the primal optimization problem using different allowable numbers of manipulated enzymes ranging from three to five, respectively. Tables 3 and 4 show the convergent solutions obtained by seven solvers in GAMS. For the case of the allowable numbers of three and four, we obtained two convergent solutions, but a Pareto optimal solution only. Tables 3 and 4 also show that the optimal solution for *n *allowable modulated enzymes includes the suggested enzyme set for *n *- 1 allowable modulated enzymes. This trend is same as that obtained by Voit and Signore resulting from the effect of experimental imprecision [28]. Finally, we could qualitatively speculate that the suggested enzymes to be modulated for both primal and resilience optimization problems are {G6PDH, PK, SERS, RPPK, DAHPS, SYN1} in decreasing order of priority.

In this metabolic system, we assume that concentrations of the seven co-metabolites are constant in the mathematical model. These adenine nucleotides stoichiometrically join in all of the metabolic networks of a living cell. In practical, they actively participate in the dynamics of the network and cannot be ignored. The value of the adenylate energy charges in *E. coli *cells and the ratios of [*nad*]/[*nadh*] and [*nad*]/[*nadh*] in the redox metabolic network are equal to the ratios of their basal levels and can be considered as constants [29]. Their relationships are expressed as

(2) $$\begin{array}{lll} \frac{\left[atp\right]+0.5\left[adp\right]}{\left[atp\right]+\left[adp\right]+\left[amp\right]} & =\frac{{\left[atp\right]}_{basal}+0.5{[adp]}_{basal}}{{\left[atp\right]}_{basal}+{\left[adp\right]}_{basal}+{\left[amp\right]}_{basal}}\; & \text{(1)}\\ & =c_{1},\; & \text{(2)}\\ & \; & \text{(3)} \end{array}$$

(3) $$\frac{\left[nadh\right]}{\left[nadh\right]+\left[nad\right]}=\frac{{\left[nadh\right]}_{basal}}{{\left[nadh\right]}_{basal}+{\left[nad\right]}_{basal}}=c_{2},$$

(4) $$\frac{\left[nadph\right]}{\left[nadph\right]+\left[nadp\right]}=\frac{{\left[nadph\right]}_{basal}}{{\left[nadph\right]}_{basal}+{\left[nadp\right]}_{basal}}=c_{3},$$

where *c_i _*are constants. This study also evaluated the effects of the assumption of constant co-metabolite concentrations by applying equations (2)-(4) to the optimization problems. Similar procedures were also used to obtain the optimal modulations for the primal and resilience optimization problems. The computational results can be found in Tables S1 and S2 of Additional File 3. We could conclude that the improved flux ratios are a little different from those obtained from the optimization problems with constant co-metabolite concentrations. The order of priority of the suggested modulated enzymes is nearly identical to that obtained based on the assumption of constant co-metabolite concentrations except when the number of allowable modulated enzymes is five.

## Conclusions

The optimization of biological systems, which is a branch of metabolic engineering, has generated a lot of industrial and academic interest for a long time. The ultimate goal of this optimization is to find the optimal mutation strategy for improving productivity. Model-based optimization strategies have been applied to analyze and design metabolic networks during the last decade. The accuracy of optimization results depends heavily on the development of essential kinetic models of metabolic networks. Kinetic models can quantitatively capture the experimentally observed regulation data of metabolic systems and are often used to find the optimal manipulation of external inputs. To address the issues of optimizing the regulatory structure of metabolic networks, it is necessary to consider qualitative effects, e.g., the resilience phenomena and cell viability constraints. Combining the qualitative and quantitative descriptions for metabolic networks makes it possible to design a viable strain and accurately predict the maximum possible flux rates of desired products.

This study introduces a generalized fuzzy multi-objective optimization approach to determine the optimal enzymatic manipulations for metabolic network systems to obtain the maximal flux ratios of desired metabolites of interest. The goal of this optimization problem is to find the maximal synthesis rates of desired metabolites and the minimum set of manipulated enzymes simultaneously based on kinetic models. The kinetic model was directly used for the optimization problem and MINLP solvers were applied to determine which enzymes to manipulate and how their corresponding activities changed. This study applies fuzzy equal and fuzzy inequality operations to the optimization problem to deal with the resilience phenomena and cell viability constraints. This study tests the practical utility of the proposed approach by applying it to two metabolic networks of *S. cerevisiae *and *E. coli*. The resulting optimal enzymatic manipulations for metabolic networks and the maximum flux ratios for desired metabolites are more justifiable based on biological knowledge. We could qualitatively speculate the priority of modulated enzymes obtained by iteratively solving the optimization problems using various allowable numbers of manipulated enzymes. These results can help microbiologists make a proper decision when genetically modifying a microorganism.

## Methods

### Kinetic model

The dynamics of a metabolic network can be represented generically using a set of nonlinear ordinary differential equations with the following structure:

(5) $$\frac{dx}{dt}=Sv\left(x,e;\theta \right),$$

where **x **∈ ℝ*^n ^*is a vector of concentrations of metabolites or pools, **e **∈ ℝ*^m ^*is a vector of enzyme levels corresponding to the enzyme activities, *θ *∈ ℝ*^p ^*is a vector of parameters, **v **∈ ℝ*^m ^*is a vector of reaction rates, and *S *∈ ℝ*^n×m ^*is the stoichiometric matrix describing the interconnecting fluxes. The stoichiometry of biochemical reactions is constant. Kinetic aspects are used to capture the dynamics of a system and may change rather quickly as they are driven by the state of the system. The stoichiometry of a biochemical pathway determines the wiring diagram of the network, describes which fluxes enter or leave which pool, and ensures that mass is conserved in the process. The reaction rate can be expressed by the power-law functions or Michaelis-Menten-based rate laws in the field of biological systems.

### Primal optimization problem

The model outlined above can be combined with mathematical programming to support microbiologists in the biotechnological improvement of microorganisms in industrial applications [9]. The optimization problem can consider many objectives. For example, Sorribas et al. discussed many criteria used in evolution problems [23]. The current study uses multi-objective optimization approaches to deal with the following issues; What is the minimum set of enzymes in a given microorganism that should be modulated to maximize the synthesis flux ratios of the desired end products, simultaneously? How to make a proper decision when genetically modifying a microorganism if we find that more than one biological response should be optimized? The multi-objective optimization problem is generally expressed as follows:

(6) $$\underset{e,x,y}{\max}\frac{v_{i}}{v_{i}^{basal}},i\in \Sigma_{O},$$

(7) $$\underset{e,x,y}{\min}\overset{m}{\underset{j=1}{\sum }}y_{j},$$

where $v_{i}^{basal}$ is the basal value of the *i^th ^*flux *v_i _*∈ **v**, Σ*_O _*∈ ℕ*^r ^*is the set of indices of production rates to be maximized, *r *is the number of target fluxes to be maximized, and the binary variable *y_j _*∈ **y **indicates whether the *j^th ^*enzyme should be modulated and is defined as

$$y_{j}=\left\{\begin{array}{cc} 1 & if\;enzyme\;j\;is\;modulated,\\ 0 & otherwise, \end{array}\right)$$

Equation (6) is a general formula for simultaneously maximizing a set of metabolite synthesis rates. Several researchers have introduced genetic manipulations to redistribute various metabolic fluxes in a metabolic network to enhance the desired synthesis rates [16,27]. Equation (7) obtains the minimum set of modulated enzymes in the metabolic network.

This study includes the following additional constraints for each enzyme in the metabolic networks.

(8) $$\begin{array}{c} \left(y_{i}e_{i}^{LB}+\left(1-y_{i}\right)b_{i}^{LB}\right)\leq e_{i}\leq \left(y_{i}e_{i}^{UB}+\left(1-y_{i}\right)b_{i}^{UB}\right),\\ i=1,\ldots ,m, \end{array}$$

(9) $$\overset{m}{\underset{i=1}{\sum }}y_{i}\geq 1,$$

where $e_{i}^{LB}$ and $e_{i}^{UB}$ are the lower and upper bounds for each significant modulated enzyme, and $b_{i}^{LB}$ and $b_{i}^{UB}$ are the lower and upper perturbation bounds for each non-significant enzyme. When the lower and upper factors for significant and non-significant enzymes and their basal value $e_{i}^{basal}$ are given by users, these bounds can be evaluated as follows: $e_{i}^{LB}=\gamma_{e_{i}}^{LB}e_{i}^{basal}$, $e_{i}^{UB}=\gamma_{e_{i}}^{UB}e_{i}^{basal}$, $b_{i}^{LB}=\gamma_{b_{i}}^{LB}e_{i}^{basal}$, and $b_{i}^{UB}=\gamma_{b_{i}}^{UB}e_{i}^{basal}$, where the lower significant and non-significant factors, $\gamma_{e_{i}}^{LB}$ and $\gamma_{b_{i}}^{LB}$, are less than one, and the upper factors, $\gamma_{e_{i}}^{UB}$ and $\gamma_{b_{i}}^{UB}$, are greater than one. In addition, the lower non-significant factor $\gamma_{b_{i}}^{LB}$ must be greater than $\gamma_{e_{i}}^{LB}$ and the upper non-significant factor $\gamma_{b_{i}}^{UB}$ must be smaller than $\gamma_{e_{i}}^{UB}$. Constraint (8) provides the lower and upper bounds of each enzyme. If enzyme *i *is not modulated, then its activity can have a small variance around its basal value $e_{i}^{basal}$. The lower and upper perturbation bounds, $b_{i}^{LB}$ and $b_{i}^{UB}$, restrict the activity variance of *i^th ^*un-modulated enzyme due to other enzyme alterations. A similar non-significant variance in enzyme activity discussed in the assumption of ROOM [18]. Constraint (9) indicates that at least one enzyme/gene should be manipulated. The concentration for each metabolite is restricted by its lower and upper bounds,

(10) $$\gamma_{x_{i}}^{LB}x_{i}^{basal}\leq x_{i}\leq \gamma_{x_{i}}^{UB}x_{i}^{basal},i=1,\ldots ,n,$$

where $\gamma_{x_{i}}^{LB}$ and $\gamma_{x_{i}}^{UB}$ are the lower and upper bounded factors for each metabolite, respectively, and $x_{i}^{basal}$ is the basal value of the *i*^th ^metabolite *x_i _*∈ **x**.

An abnormally high protein or intermediate concentration in a metabolic system renders a cell non-viable. This is because the burden on cellular metabolism is too high for the cell to survive or the cellular osmolarity constraint is violated. Several researchers have introduced a constraint on the total enzyme concentration to overcome this issue and assure that it never reaches a value that is considered unacceptable for the cell viability [1,6,10,13]. Moreover, the cell viability and optimal synthesis rates effectively limit the total intermediate metabolite concentration. The total metabolite and enzyme concentration constraints for cell viability are expressed as follows:

(11) $$\overset{n}{\underset{i=1}{\sum }}x_{i}\leq \zeta_{x}\overset{n}{\underset{i=1}{\sum }}x_{i}^{basal},$$

(12) $$\overset{m}{\underset{i=1}{\sum }}e_{i}\leq \zeta_{e}\overset{m}{\underset{i=1}{\sum }}e_{i}^{basal},$$

where ζ*_x _*and ζ*_e _*are the restriction factors for the constraints on total metabolite concentration and total enzyme concentration, respectively.

The primal multi-objective optimization problem formulated by equations (5) to (12) is a multi-objective mixed-integer nonlinear programming problem. Many methods are capable of solving multi-objective optimization problems (MOOPs) to obtain the Pareto front [30-32] and generally fall into one of two categories: generating methods and preference-based methods. Each method has its advantages and disadvantages, as discussed in several articles [30-32]. Generating methods can apply a scalarization approach to convert an MOOP into a single-objective optimization problem (SOOP) with different factors to find one Pareto optimal solution. A series of the SOOP with various factors must be solved to find a Pareto front of the MOOP. Evolutionary algorithms can be directly applied to the MOOP to find the Pareto front, but they are time-consuming. A decision maker (DM) then selects a desired solution from the Pareto front. In contrast, the preference-based methods require preferences in advance from the DM and then find a satisfactory solution. However, preferences are generally difficult to specify with limited knowledge of the values of objective functions. Therefore, an interactive algorithm must be carried out to find a compromised solution.

The original *ε*-constraint, one of the generating methods, retains only one of the objective functions as the criterion and converts the others into inequality constraints. This approach is suitable for the MOOP with objective functions, which can easily assign the *ε*-values. The weighted infinite norm method is a reference-goal method that can conveniently determine a trade-off solution if the lower and upper bounds of each objective value are known in advance. This study combines the *ε*-constraint method and weighted infinite norm method to solve the primal multi-objective optimization problem. The objective function in equation (7) can be straightforwardly converted into an *ε*-constraint because the number of enzymes is an integer value. The primal multi-objective optimization problem can be transformed into a weighted infinite norm problem defined as follows:

(13) mine,x,y∈Ω maxi∈ΣOviUB-viviUB-viLB,

subject to

(14) $$\overset{m}{\underset{j=1}{\sum }}y_{j}\leq \varepsilon ,$$

where the user provides the allowable number *ε *of the manipulated enzymes in advance, the lower bound $v_{i}^{LB}$ is equal to its basal flux $v_{i}^{basal}$, the upper bound $v_{i}^{UB}$ can be estimated by SOOP that maximizes *v_i _*only, and the feasible set Ω consists of all feasible solutions that satisfy the material balance equations in the steady state and the constraints in equations (8)-(12).

### Resilience phenomena

A strain may reflect resilience phenomena after genetic interventions. This study introduces fuzzy equal and fuzzy max operators to deal with the resilience phenomena and the maximization of target fluxes, respectively. The minimization of the number of enzymes to be modulated is still defined on a crisp domain. The primal multi-objective optimization problem is therefore extended to be a generalized fuzzy multi-objective optimization problem (GFMOOP) that can be expressed as follows:

(15) $$\tilde{\underset{e,x,y}{\max}}\frac{v_{i}}{v_{i}^{basal}}=\tilde{\underset{e,x,y}{\max}}f_{i}≿\left[f_{i}^{LB},f_{i}^{UB}\right],i\in \Sigma_{O},$$

(16) $$\tilde{\underset{e,x,y}{\text{equal}}}\left(x_{j}\approx x_{j}^{basal}\right),j\in \Sigma_{X},$$

(17) $$\tilde{\underset{e,x,y}{\text{equal}}}\left(e_{k}\approx e_{k}^{basal}\right),k\in \Sigma_{E},$$

(18) $$\underset{e,x,y}{\min}\overset{m}{\underset{j=1}{\sum }}y_{j},$$

where Σ*_X _*∈ ℕ*^n ^*is the set of metabolite indices and Σ*_E _*∈ ℕ*^m ^*is the set of enzyme indices. Here, the symbols, ≿ and ≈ denote a relaxed or fuzzy version of the ordinary inequality "≥" and equality "=", respectively. The fuzzy maximization, "$\tilde{\max}$", in equation (15) means that the enzyme manipulation is completely acceptable if the *i^th ^*flux ratio exceeds its upper bound $f_{i}^{UB}$, which can be estimated from the previous primal optimization problem. Conversely, the design is completely unacceptable if the *i^th ^*flux ratio is less than the lower bound $f_{i}^{LB}$. The lower bound is generally equal to one, meaning that the modified flux should exceed its basal value. Equations (16) and (17) are "fuzzy equal $\left(\tilde{equal}\right)$" objective functions that represent the fuzzy goals. For example, the metabolite concentration *x_j _*and enzyme activity *e_k _*should be restored to a state that is as close to the wild-type as possible. Equation (18) is the crisp objective function as same as Equation (7).

The cell viability constraints in equations (11) and (12) are crisp limits, indicating that all cells die when any of the inequality constraints is violated. These constraints are not so strict in practical situations according to the growth patterns and kinetics of cells in culture [33]. In general, cells can survive when each total amount of metabolites and enzymes is within a wide interval over the critical value. This study applies the fuzzy inequality constraint to handle this practical situation. In this case, the restrictions for cell viability are softened as follows:

(19) $$\overset{n}{\underset{i=1}{\sum }}x_{i}≾\left[\zeta_{x}^{LB},\zeta_{x}^{UB}\right]\overset{n}{\underset{i=1}{\sum }}x_{i}^{basal},$$

(20) $$\overset{m}{\underset{i=1}{\sum }}e_{i}≾\left[\zeta_{e}^{LB},\zeta_{e}^{UB}\right]\overset{m}{\underset{i=1}{\sum }}e_{i}^{basal},$$

where the symbol "≾" denotes a fuzzy version of the ordinary inequality "≤". Here, $\zeta_{x∕e}^{LB}$ and $\zeta_{x∕e}^{UB}$ are the lower and upper restriction factors for the fuzzy constraints on total metabolite/enzyme concentrations, respectively. The interval bound $\left[\zeta_{x∕e}^{LB},\zeta_{x∕e}^{UB}\right]$ indicates that the microbes have some degree of satisfaction if each total metabolite/enzyme concentration is within its boundary. The lower bounds of the fuzzy inequality constraints mean that the microbes are completely survival if both total metabolite/enzyme concentration constraints in equations (19) and (20) are less than their lower limits. Conversely, the microbes completely die if one of the total metabolite/enzyme concentration constraints exceeds its upper limit. This situation indicates that the solution is infeasible.

### Goal-attainment problem

The objective functions of GFMOOP are defined on the fuzzy and crisp domains. Almost no studies discuss how to obtain an optimal Pareto solution of the GFMOOP. This study converts the GFMOOP into a fuzzy multi-objective optimization problem with *ε*-constraints, abbreviated as *ε*-FMOOP, by transforming the crisp objective function to an *ε*-constraint. To solve the *ε*-FMOOP, each fuzzy objective functions for maximizing synthesis rate can be quantified by eliciting the following membership function:

(21) $$\eta_{i}\left(f_{i}\right)=\left\{\begin{array}{cc} 0 & f_{i}^{LB}>f_{i},\\ d_{i} & f_{i}^{LB}\leq f_{i}\leq f_{i}^{UB},\\ 1 & f_{i}>f_{i}^{UB},i\in \Sigma_{O}, \end{array}\right)$$

where *d_i _*is a strictly monotonically increasing function for evaluating the degree of satisfaction. The maximum synthesis rate becomes somewhat acceptable if its objective value lies between the lower and upper bounds. The membership function value is zero if the synthesis rate is less than the desired lower bound. Conversely, the grade of membership function is one when the synthesis rate exceeds its upper bound.

The membership function for each fuzzy equal objective function in equation (16) is defined as follows:

(22) $$\eta_{j}\left(x_{j}\right)=\left\{\begin{array}{cc} 0 & x_{j}^{LB}>x_{j},\\ d_{j}^{\prime } & x_{j}^{LB}\leq x_{j}\leq x_{j}^{basal},\\ 1 & x_{j}=x_{j}^{basal},\\ d_{j}^{\prime \prime } & x_{j}^{basal}\leq x_{j}\leq x_{j}^{UB},\\ 0 & x_{j}>x_{j}^{UB},j\in \Sigma_{X}, \end{array}\right)$$

where $x_{j}^{LB}$ and $x_{j}^{UB}$ are the lower and upper bounds for the concentration of the *j^th ^*metabolite, and the user-provided functions $d_{j}^{\prime }$ and $d_{j}^{\prime \prime }$ are strictly monotonically increasing and decreasing functions, respectively.

The membership function value is zero if the metabolite concentration exceeds the bounded interval. When the metabolite concentration lies within the bounds, the metabolite concentration should be as close as possible to its basal value. The membership function for the fuzzy equal objective function for each enzyme in equation (17) can be defined as equation (22). The fuzzy inequality constraint in equation (19) can be quantified by eliciting the following membership function:

(23) $$\eta_{k}\left(\overset{n}{\underset{i=1}{\sum }}x_{i}\right)=\left\{\begin{array}{cc} 1 & \zeta_{x}^{LB}\overset{n}{\underset{i=1}{\sum }}x_{i}^{basal}>\overset{n}{\underset{i=1}{\sum }}x_{i},\\ d_{k}^{{}^{\prime \prime \prime }} & \zeta_{x}^{LB}\overset{n}{\underset{i=1}{\sum }}x_{i}^{basal}\leq \overset{n}{\underset{i=1}{\sum }}x_{i}\leq \zeta_{x}^{UB}\overset{n}{\underset{i=1}{\sum }}x_{i}^{basal},\\ 0 & \overset{n}{\underset{i=1}{\sum }}x_{i}>\zeta_{x}^{UB}\overset{n}{\underset{i=1}{\sum }}x_{i}^{basal}, \end{array}\right)$$

where $d_{k}^{\prime \prime \prime }$ is a strictly monotonically decreasing function for evaluating the degree of satisfaction. The value of the membership function is one if the total amount of the metabolite concentrations is less than the desired lower bound. Conversely, the grade of membership function is zero when the total amount of the metabolite concentrations exceeds its upper bound. The cell viability becomes somewhat acceptable if the total amount lies between the bounded interval.

**Figure 5:**
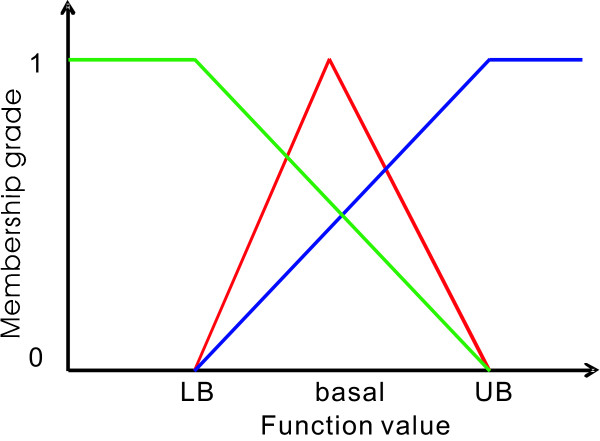
**Membership functions for fuzzy objective function, fuzzy equal objective, and fuzzy inequality constraint**. Membership functions for fuzzy maximization objective function (blue line), fuzzy equality function (red line), and fuzzy inequality function (green line).

Sakawa proposed five types of membership functions to evaluate membership grades: linear, exponential, hyperbolic, inverse, and piecewise linear functions [31]. For concise illustration, this study uses linear membership functions to formulate each fuzzy objective function, fuzzy equal objective, and fuzzy inequality constraint (see Figure 5). Each membership level is between zero and one. The zero level indicates that the user is completely unsatisfied with the corresponding goal. In contrast, the grade is one if the value for each goal is 100% satisfactory. Figure 5 illustrates the relationships for all membership functions. The fuzzy optimization finds a compromised solution from these goals. The membership grade is zero for every fuzzy objective and every fuzzy equal objective and one for every fuzzy inequality constraint when the values of fuzzy objective functions, fuzzy equal objectives, and fuzzy inequality constraints are less than their lower bounds. The intersection for these membership functions is zero. Conversely, if the values of fuzzy objective functions, fuzzy equal objectives, and fuzzy inequality constraints are greater than the upper bounds, the intersection for these membership functions is still zero. The aim of fuzzy optimization is to find a maximum intersection for all membership functions between the desired boundaries.

Having elicited the membership functions for fuzzy objective functions, fuzzy equal objectives, and fuzzy inequality constraints, the *ε*-FMOOP can be expressed as the goal attainment problem:

(24) $$\underset{e,x,y}{\min}\;\eta_{D}=\underset{e,x,y}{\min}\left\{\underset{i\in \Sigma }{\max}\left[{\bar{\eta }}_{i}-\eta_{i}\left(f_{i}\right)\right]+\delta \underset{i\in \Sigma }{\sum }\left[{\bar{\eta }}_{i}-\eta_{i}\left(f_{i}\right)\right]\right\},$$

where ${\bar{\eta }}_{i}$ is the ideal preferred goal, Σ = Σ*_O _*∪ Σ*_X _*∪ Σ*_E_*, and *η_D _*denotes an aggregation function defined on the crisp domain Ω, which consists of the feasible solutions satisfied equation (5), the crisp bounds in equations (8)-(9), and the *ε*-constraint in equation (14). Sakawa introduced several aggregation functions in which the value of the aggregation function can be interpreted as an overall degree of satisfaction with user's fuzzy goals [31]. This study uses the first term of the aggregation function in the brace of equation (24) to identify the optimal trade-off solution that is nearest to the ideal preferred goal, ${\bar{\eta }}_{i}$, which indicates 100% satisfaction. The second term avoids testing the uniqueness for optimality of the solution and the constant *δ *is a small positive value within 10^-3 ^- 10^-5^. The fuzzy goal attainment approach can directly find a satisfactory solution in the Pareto set without yielding the Pareto frontier of the problem.

## Competing interests

The authors declare that they have no competing interests.

## Authors' contributions

WHW implemented the optimization methods, performed the computational experiments, and contributed to the analysis of the experimental data. FSW conceived of the study, participated in its design and coordination, and analyzed the results. WHW and FSW wrote the manuscript. MSC assisted in developing the optimization methods and finalizing the manuscript. All authors read and approved the final manuscript.

## Supplementary Material

Additional file 1 **Mathematical model of anaerobic fermentation in *S. cerevisiae***. This file includes the set of differential equations and rate equations for anaerobic fermentation in *S. cerevisiae*, all of the relevant definitions of state and independent variables, and the nominal values of parameters appearing in the differential equations. Click here for file

Additional file 2 **Mathematical model of central carbon metabolism in *Escherichia coli***. This file includes the set of differential equations and rate equations for central carbon metabolism in *E. coli *and all of the nominal values of parameters appearing in the differential equations. Click here for file

Additional file 3 **Results of multi-synthesis maximization by *Escherichia coli *considering energy and redox conservation for co-metabolites**. This file includes the results of multi-synthesis maximization by *Escherichia coli *under the conservation of co-metabolites. The suggested modulated enzymes and the optimal synthesis rates considering resilience phenomena or not are shown for comparison. Click here for file
