# Supplementary material for: Multi-objective optimization of enzyme manipulations in metabolic networks considering resilience effects
Source: BMC Syst Biol. 2011 Sep 19;5:145. doi: 10.1186/1752-0509-5-145 (PMC3203348; doi:10.1186/1752-0509-5-145)
Supplement: Additional file 1 — Mathematical model of anaerobic fermentation in S. cerevisiae. This file includes the set of differential equations and rate equations for anaerobic fermentation in S. cerevisiae, all of the relevant definitions of state and independent variables, and the nominal values of parameters appearing in the differential equations. [file 1752-0509-5-145-S1.PDF]

Supplementary information
Multi-objective optimization of enzyme manipulations in metabolic networks considering resilience effects

Wu-Hsiung Wu[1], Feng-Sheng Wang*[2] and Maw-Shang Chang[1]

[1]Department of Computer Science and Information Engineering, National Chung Cheng University, Chiayi 62102, Taiwan
[2]Department of Chemical Engineering, National Chung Cheng University, Chiayi 62102, Taiwan

Email: Wu-Hsiung Wu - wwh@cs.ccu.edu.tw; Feng-Sheng Wang*- chmfsw@ccu.edu.tw; Maw-Shang Chang - mschang@cs.ccu.edu.tw;

*Corresponding author

Mathematical model of anaerobic fermentation in *S. cerevisiae*

Saccharomyces cerevisiae is the most commonly used yeast, which is well studied and widely used in industrial application. The reasons for selecting this metabolic systems are that ethanol fermentation using *S. cerevisiae* is a well established technology and the detailed knowledge about the glucose uptake rate and the formation rate of ethanol in *S. cerevisiae* is available. This GMA model was developed by Curto et al. [1] for analyzing the anaerobic ethanol fermentation of *S. cerevisiae* at steady state. It consists of 5 nonlinear ordinal differential equations and 8 nonlinear rate equations. The maximum reaction rates can be found from an online model database (JWS).

Model equations

$$
\begin{aligned}
\frac{\mathrm{d}[glc]}{\mathrm{d}t} &= v_{HXT} - v_{GLK} \\
\frac{\mathrm{d}[g6p]}{\mathrm{d}t} &= v_{GLK} - v_{PFK} - v_{TPS} \\
\frac{\mathrm{d}[f6p]}{\mathrm{d}t} &= v_{PFK} - v_{TDH} - \frac{1}{2}v_{GOL} \\
\frac{\mathrm{d}[pep]}{\mathrm{d}t} &= 2v_{TDH} - v_{PYK} \\
\frac{\mathrm{d}[atp]}{\mathrm{d}t} &= 2v_{TDH} + v_{PYK} - v_{GLK} - v_{TPS} - v_{PFK} - v_{ATPase}
\end{aligned}
$$

Rate equations

The rate equations are represented as the following power-law form:

$$v_{HXT} = 0.8122[g6p]^{-0.2344}[HXT]$$

$$v_{GLK} = 2.8674[glc]^{0.7464}[atp]^{0.0243}[GLK]$$

$$v_{PFK} = 0.5228[g6p]^{0.7318}[atp]^{-0.3941}[PFK]$$

$$v_{TDH} = 0.076029[f6p]^{0.6159}[atp]^{0.1308}[TDH]$$

$$v_{PYK} = 0.09471[f6p]^{0.05}[pep]^{0.533}[atp]^{-0.0822}[PYK]$$

$$v_{TPS} = 0.000904[g6p]^{8.6107}[TPS]$$

$$v_{GOL} = 0.09272[f6p]^{0.05}[pep]^{0.533}[atp]^{-0.0822}[GOL]$$

$$v_{ATPase} = [atp][ATPase]$$

where each reaction is denoted as follows: v_{HXT} (sugar transport system), v_{GLK} (hexokinase), v_{PFK} (phosphofructokinase), v_{TPS} (glycogen synthetase), v_{TDH} (glyceraldehyde 3-phosphate dehydrogenase), v_{GOL} (glycerol 3-phosphate dehydrogenase, proportional to PYK), v_{PYK} (pyruvate kinase),and v_{ATPase} (ATPase). The rate of ethanol production is given directly by the flux through the pyruvate kinase reaction. State variables in the model are the concentrations of internal glucose $[glc]$, glucose-6-phosphate $[g6p]$, fructose-1,6-diphosphate $[f6p]$, phosphoenol pyruvate $[pep]$, and ATP $[atp]$. Independent variables with constant values are: effective hexose transport $[HXT]$, hexokinase/glucokinase $[GLK]$, phosphofructokinase $[PFK]$, glyceraldehyde dehydrogenase $[TDH]$, pyruvate kinase $[PYK]$, glycogen and trehalose production $[TPS]$, glycerol production $[GOL]$, and ATPase $[ATPase]$.

References

1. Curto R, Sorribas A, Cascante M: **Comparative characterization of the fermentation pathway of *Saccharomyces cerevisiae* using biochemical systems theory and metabolic control analysis: Model definition and nomenclature**. *Mathematical Biosciences* 1995, **130**:25–50.
