# Supplementary material for: Multi-objective optimization of enzyme manipulations in metabolic networks considering resilience effects
Source: BMC Syst Biol. 2011 Sep 19;5:145. doi: 10.1186/1752-0509-5-145 (PMC3203348; doi:10.1186/1752-0509-5-145)
Supplement: Additional file 2 — Mathematical model of central carbon metabolism in Escherichia coli. This file includes the set of differential equations and rate equations for central carbon metabolism in E. coli and all of the nominal values of parameters appearing in the differential equations. [file 1752-0509-5-145-S2.PDF]

Supplementary information
Multi-objective optimization of enzyme manipulations in metabolic networks considering resilience effects

Wu-Hsiung Wu[1], Feng-Sheng Wang*[2] and Maw-Shang Chang[1]

[1]Department of Computer Science and Information Engineering, National Chung Cheng University, Chiayi 62102, Taiwan
[2]Department of Chemical Engineering, National Chung Cheng University, Chiayi 62102, Taiwan

Email: Wu-Hsiung Wu - wwh@cs.ccu.edu.tw; Feng-Sheng Wang*- chmfsw@ccu.edu.tw; Maw-Shang Chang - mschang@cs.ccu.edu.tw;

*Corresponding author

Mathematical model of central carbon metabolism in *Escherichia coli*

The central carbon metabolism plays essential roles in many organisms, such as *E. coli* and *C. glutamicum*, providing energy metabolism and precursors for aromatic amino acids and serine syntheses. The central carbon metabolism of *E. coli* is a complex network. Chassagnole et al. [1] developed a nonlinear dynamic model for part of central carbon metabolism of *E. coli*. In addition to the ability to describe the experimentally observed dynamic behavior of metabolites in metabolic networks, the model is also capable of describing the intracellular metabolite oscillations observed in experiments with *E. coli* [2]. This model links the kinetics of sugar transporter PTS (phosphor-transferase system) with glycolysis and pentose-phosphate pathways and is used to support the exploration of the central carbon metabolism of *E. coli*. It consists of 18 nonlinear ordinal differential equations and 30 nonlinear rate equations. Seven co-metabolites (*amp*, *adp*, *atp*, *nad*, *nadh*, *nadp* and *nadph*) are included in the model and their concentrations are assumed to be constant. The maximum reaction rates can be found from an online model database (JWS).

Model equations

The mathematical model of central Carbon metabolism in *Escherichia coli* is described by the following set of ordinal differential equations:

$$\frac{\mathrm{d}[glc]}{\mathrm{d}t} = 2.778 \times 10^{-5}(111.1 - [glu]) - 0.0154 v_{PTS}$$
$$\frac{\mathrm{d}[g6p]}{\mathrm{d}t} = v_{PTS} - v_{PGI} - v_{G6PDH} - v_{PGM} - \mu[g6p]$$
$$\frac{\mathrm{d}[f6p]}{\mathrm{d}t} = v_{PGI} + v_{TKB} + v_{TA} - v_{PFK} - 2v_{MURS} - \mu[f6p]$$
$$\frac{\mathrm{d}[fdp]}{\mathrm{d}t} = v_{PFK} - v_{ALDO} - \mu[fdp]$$
$$\frac{\mathrm{d}[gap]}{\mathrm{d}t} = v_{ALDO} + v_{TIS} + v_{TKA} + v_{TKB} + v_{TRPS} - v_{GAPDH} - v_{TA} - \mu[gap]$$
$$\frac{\mathrm{d}[dhap]}{\mathrm{d}t} = v_{ALDO} - v_{TIS} - v_{G3PDH} - \mu[dhap]$$
$$\frac{\mathrm{d}[pgp]}{\mathrm{d}t} = v_{GAPDH} - v_{PGK} - \mu[pgp]$$
$$\frac{\mathrm{d}[3pg]}{\mathrm{d}t} = v_{PGK} - v_{PGluMu} - v_{SERS} - \mu[3pg]$$
$$\frac{\mathrm{d}[2pg]}{\mathrm{d}t} = v_{PGluMu} - v_{ENO} - \mu[2pg]$$
$$\frac{\mathrm{d}[pep]}{\mathrm{d}t} = v_{ENO} - v_{PK} - v_{PTS} - v_{PEPC} - v_{DAHPS} - v_{SYN1} - \mu[pep]$$
$$\frac{\mathrm{d}[pyr]}{\mathrm{d}t} = v_{PK} + v_{PTS} + v_{TRPS} + v_{METS} - v_{PDH} - v_{SYN2} - \mu[pyr]$$
$$\frac{\mathrm{d}[6pg]}{\mathrm{d}t} = v_{G6PDH} - v_{PGDH} - \mu[6pg]$$
$$\frac{\mathrm{d}[ribu5p]}{\mathrm{d}t} = v_{PGDH} - v_{RU5P} - v_{R5PI} - \mu[ribu5p]$$
$$\frac{\mathrm{d}[xyl5p]}{\mathrm{d}t} = v_{RU5P} - v_{TKA} - v_{TKB} - \mu[xyl5p]$$
$$\frac{\mathrm{d}[sed7p]}{\mathrm{d}t} = v_{TKA} - v_{TA} - \mu[sed7p]$$
$$\frac{\mathrm{d}[rib5p]}{\mathrm{d}t} = v_{R5PI} - v_{TKA} - v_{RPPK} - \mu[rib5p]$$
$$\frac{\mathrm{d}[e4p]}{\mathrm{d}t} = v_{TA} - v_{TKB} - v_{DAHPS} - \mu[e4p]$$
$$\frac{\mathrm{d}[g1p]}{\mathrm{d}t} = v_{PGM} - v_{G1PAT} - \mu[g1p]$$

Rate equations

The rate equations are represented as follows:

$$v_{PFK} = \frac{v_{max}^{PFK}[atp][f6p]}{\left([atp] + 0.123\left(1 + \frac{[adp]}{4.14}\right)\right)\left([f6p] + \frac{0.325A}{B}\right)\left(1 + \frac{5629067}{\left(1+\frac{[f6p]B}{0.325A}\right)^{11.1}}\right)}$$

$$A = 1 + \frac{[pep]}{3.26} + \frac{[adp]}{3.89} + \frac{[amp]}{3.2}$$

$$B = 1 + \frac{[adp]}{128} + \frac{[amp]}{19.1}$$

$$v_{PK} = \frac{v_{max}^{PK}[pep][adp]\left(1 + \frac{[pep]}{0.31}\right)^3}{0.31\left(1000\left(\frac{1+\frac{[atp]}{22.5}}{1+\frac{[fdp]}{0.19}+\frac{[amp]}{0.2}}\right)^4 + \left(1 + \frac{[pep]}{0.31}\right)^4\right)(0.26 + [adp])}$$

$$v_{PTS} = \frac{v_{max}^{PTS}\frac{[glu][pep]}{[pyr]}}{\left(3082.3 + \frac{0.01[pep]}{[pyr]} + 245.3[glu] + \frac{[glu][pep]}{[pyr]}\right)\left(1 + \frac{[g6p]^{3.66}}{2.15}\right)}$$

$$v_{PGI} = \frac{v_{max}^{PGI}\left([g6p] - \frac{[f6p]}{0.1725}\right)}{2.9\left(1 + \frac{[f6p]}{0.266\left(1+\frac{[6pg]}{0.2}\right)} + \frac{[6pg]}{0.2}\right) + [g6p]}$$

$$v_{ALDO} = \frac{v_{max}^{ALDO}\left([fdp] - \frac{[gap][dhap]}{0.144}\right)}{1.75 + [fdp] + 0.306[dhap] + 0.306[gap] + \frac{[fdp][gap]}{0.6} + \frac{[gap][dhap]}{0.288}}$$

$$v_{GAPDH} = \frac{v_{max}^{GAPDH}\left([gap][nad] - \frac{[pgp][nadh]}{0.63}\right)}{\left(0.683\left(1 + \frac{[pgp]}{1.04\times10^{-5}}\right) + [gap]\right)\left(0.252\left(1 + \frac{[nadh]}{1.09}\right) + [nad]\right)}$$

$$v_{PGK} = \frac{v_{max}^{PGK}\left([adp][pgp] - \frac{[atp][3pg]}{1934.4}\right)}{\left(0.185\left(1 + \frac{[atp]}{0.653}\right) + [adp]\right)\left(0.0468\left(1 + \frac{[3pg]}{0.473}\right) + [pgp]\right)}$$

$$v_{G6PDH} = \frac{v_{max}^{G6PDH}[g6p][nadp]}{(14.4 + [g6p])\left(1 + \frac{[nadph]}{6.43}\right)\left(0.0246\left(1 + \frac{[nadph]}{0.01}\right) + [nadp]\right)}$$

$$v_{PGDH} = \frac{v_{max}^{PGDH}[6pg][nadp]}{(37.5 + [6pg])\left(0.0506\left(1 + \frac{[nadph]}{0.0138}\right)\left(1 + \frac{[atp]}{208}\right) + [nadp]\right)}$$

$$v_{TIS} = \frac{v_{max}^{TIS}\left([dhap] - \frac{[gap]}{1.39}\right)}{2.8\left(1 + \frac{[gap]}{0.3}\right) + [dhap]}$$

$$v_{PGluMu} = \frac{v_{max}^{PGluMu}\left([3pg] - \frac{[2pg]}{0.188}\right)}{0.2\left(1 + \frac{[2pg]}{0.369}\right) + [3pg]}$$

$$v_{ENO} = \frac{v_{max}^{ENO}\left([2pg] - \frac{[pep]}{6.73}\right)}{0.1\left(1 + \frac{[pep]}{0.135}\right) + [2pg]}$$

$$v_{PGM} = \frac{v_{max}^{PGM}\left([g6p] - \frac{[g1p]}{0.196}\right)}{1.038\left(1 + \frac{[g1p]}{0.0136}\right) + [g6p]}$$

$$v_{PEPC} = \frac{v_{max}^{PEPC}[pep]\left(1 + \left(\frac{[fdp]}{0.7}\right)^{4.21}\right)}{4.07 + [pep]}$$

$$v_{G1PAT} = \frac{v_{max}^{G1PAT}[g1p][atp]\left(1 + \left(\frac{[fdp]}{0.119}\right)^{1.2}\right)}{(3.2 + [g1p])(4.42 + [atp])}$$

$$v_{DAHPS} = \frac{v_{max}^{DAHPS}[e4p]^{2.6}[pep]^{2.2}}{(0.035 + [e4p]^{2.6})\,(0.0053 + [pep]^{2.2})}$$

$$v_{PDH} = \frac{v_{max}^{PDH}[pyr]^{3.68}}{1159 + [pyr]^{3.68}}$$

$$v_{RPPK} = \frac{v_{max}^{RPPK}[rib5p]}{0.1 + [rib5p]}$$

$$v_{G3PDH} = \frac{v_{max}^{G3PDH}[dhap]}{1 + [dhap]}$$

$$v_{SERS} = \frac{v_{max}^{SERS}[3pg]}{1 + [3pg]}$$

$$v_{SYN1} = \frac{v_{max}^{SYN1}[pep]}{1 + [pep]}$$

$$v_{SYN2} = \frac{v_{max}^{SYN2}[pyr]}{1 + [pyr]}$$

$$v_{RU5P} = v_{max}^{RU5P}\left([ribu5p] - \frac{[xyl5p]}{1.4}\right)$$

$$v_{R5PI} = v_{max}^{R5PI}\left([ribu5p] - \frac{[rib5p]}{4}\right)$$

$$v_{TKA} = v_{max}^{TKA}\left([rib5p][xyl5p] - \frac{[sed7p][gap]}{1.2}\right)$$

$$v_{TKB} = v_{max}^{TKB}\left([e4p][xyl5p] - \frac{[f6p][gap]}{10}\right)$$

$$v_{TA} = v_{max}^{TA}\left([sed7p][gap] - \frac{[e4p][f6p]}{1.05}\right)$$

$$v_{MURS} = v_{max}^{MURS}$$

$$v_{TRPS} = v_{max}^{TRPS}$$

$$v_{METS} = v_{max}^{METS}$$

References

1. Chassagnole C, Noisommit-Rizzi N, Schmid JW, Mauch K, Reuss M: **Dynamic modeling of the central carbon metabolism of *Escherichia coli***. *Biotechnology and Bioengineering* 2002, **79**:53–73.
2. Schaefer U, Boos W, Takors R, Weuster-Botz D: **Automated Sampling Device for Monitoring Intracellular Metabolite Dynamics**. *Analytical Biochemistry* 1999, **270**:88–96.
