# Supplementary material for: Multi-objective optimization of enzyme manipulations in metabolic networks considering resilience effects
Source: BMC Syst Biol. 2011 Sep 19;5:145. doi: 10.1186/1752-0509-5-145 (PMC3203348; doi:10.1186/1752-0509-5-145)
Supplement: Additional file 3 — Results of multi-synthesis maximization by Escherichia coli considering energy and redox conservation for co-metabolites. This file includes the results of multi-synthesis maximization by Escherichia coli under the conservation of co-metabolites. The suggested modulated enzymes and the optimal synthesis rates considering resilience phenomena or not are shown for comparison. [file 1752-0509-5-145-S3.PDF]

Supplementary information
Multi-objective optimization of enzyme manipulations in metabolic networks considering resilience effects

Wu-Hsiung Wu[1], Feng-Sheng Wang*[2] and Maw-Shang Chang[1]

[1]Department of Computer Science and Information Engineering, National Chung Cheng University, Chiayi 62102, Taiwan
[2]Department of Chemical Engineering, National Chung Cheng University, Chiayi 62102, Taiwan

Email: Wu-Hsiung Wu - wwh@cs.ccu.edu.tw; Feng-Sheng Wang*- chmfsw@ccu.edu.tw; Maw-Shang Chang - mschang@cs.ccu.edu.tw;

*Corresponding author

Results of multi-synthesis maximization by *Escherichia coli* considering energy and redox conservation for co-metabolites

Table S1: **The optimal solution for multi-synthesis maximization by *E. coli* considering energy and redox conservation for co-metabolites**

ε-value	$\frac{v^*_{PEPC}}{v^{basal}_{PEPC}}$	$\frac{v^*_{SERS}}{v^{basal}_{SERS}}$	$\frac{v^*_{DAHPS}}{v^{basal}_{DAHPS}}$	Modulated enzymes	Optimal objective value η^*_D
1	1.339	1.157	1.144	PK	$0.946^{\dagger\ddagger}$
	1.376	1.165	1.367	G6PDH	0.944
2	1.298	1.611	1.779	G6PDH, SERS	0.827
	1.247	1.506	1.645	PK, SERS	$0.856^{\dagger\ddagger}$
	1.721	1.224	2.142	PK, G6PDH	$0.936^{\ddagger}$
3	1.587	1.868	2.091	G6PDH, PK, SERS	0.795
	1.439	1.837	2.052	G6PDH, SERS, RPPK	$0.802^{\dagger\ddagger}$
4	1.792	1.966	2.232	G6PDH, PK, SERS, RPPK	0.795
	1.505	2.008	2.247	G6PDH, PK, SERS, DAHPS	0.792
5	1.621	2.280	2.559	G6PDH, PK, SERS, RPPK, DAHPS	0.766
	1.883	2.148	2.398	G6PDH, PK, SERS, RPPK, SYN1	$0.790^{\ddagger}$

The optimal enzymatic modulation to maximize aromatic amino acid, serine, and oxaloacetate synthesis rates simultaneously by *E. coli* without considering cell viability constraints, but including energy and redox conservation constraints. $\gamma^{LB}_{x_i}$ and $\gamma^{LB}_{e_i}$ are set to 0.2. $\gamma^{UB}_{x_i}$ and $\gamma^{UB}_{e_i}$ are set to 5.0. The superscript * means optimal solution, ε is the number of allowed manipulated genes, superscript † denotes that the solution is not a Pareto optimal solution, and superscript ‡ indicates that the solution is obtained by solving the nonlinear programming problem in which the modulated enzymes are selected in advance.

Table S2: **The optimal solution for multi-synthesis maximization by *E. coli* considering resilience effects and energy and redox conservation for co-metabolites**

ε-value	$\frac{v^*_{PEPC}}{v^{basal}_{PEPC}}$	$\frac{v^*_{SERS}}{v^{basal}_{SERS}}$	$\frac{v^*_{DAHPS}}{v^{basal}_{DAHPS}}$	Modulated enzymes	Optimal objective value η^*_D
1	1.321	1.150	1.137	PK	$0.949^{\dagger}$
	1.353	1.157	1.331	G6PDH	0.946
2	1.262	1.538	1.685	G6PDH, SERS	0.847
	1.212	1.436	1.555	PK, SERS	$0.876^{\dagger\ddagger}$
	1.664	1.214	2.047	PK, G6PDH	$0.938^{\ddagger}$
3	1.467	1.800	2.005	G6PDH, PK, SERS	0.811
	1.363	1.701	1.881	G6PDH, SERS, RPPK	$0.834^{\dagger\ddagger}$
4	1.633	1.903	2.117	G6PDH, PK, SERS, RPPK	0.814
	1.437	1.872	2.079	G6PDH, PK, SERS, DAHPS	$0.820^{\dagger}$
5	1.538	2.109	2.351	G6PDH, PK, SERS, DAHPS, PEPCxylase	0.797
	1.513	2.057	2.288	G6PDH, PK, SERS, RPPK, DAHPS	$0.807^{\dagger\ddagger}$
	1.779	1.975	2.187	G6PDH, PK, SERS, RPPK, SYN1	$0.822^{\ddagger}$

The optimal enzymatic modulation to maximize aromatic amino acid, serine, and oxaloacetate synthesis rates simultaneously by *E. coli* considering fuzzy cell viability constraints, fuzzy metabolic and enzyme adjustments, and energy and redox conservation constraints for $[\zeta^{LB}_{x/e}, \zeta^{UB}_{x/e}] = [1.6, 2.0]$. $\gamma^{LB}_{x_i}$ and $\gamma^{LB}_{e_i}$ are set to 0.2. $\gamma^{UB}_{x_i}$ and $\gamma^{UB}_{e_i}$ are set to 5.0. The superscript * means optimal solution, ε is the number of allowed manipulated genes, superscript † denotes that the solution is not a Pareto optimal solution, and superscript ‡ indicates that the solution is obtained by solving the nonlinear programming problem in which the modulated enzymes are selected in advance.
